# Research on Image Recognition of Gymnastics Sports Injuries Based on Deep Learning

**DOI:** 10.1155/2022/8987006

**Published:** 2022-06-28

**Authors:** Peng Jia, Yixiong Xu

**Affiliations:** ^1^School of Physical Education, Jiangxi Normal University, Nanchang 330022, China; ^2^School of Physical Education, Nanchang Normal University, Nanchang 330032, China

## Abstract

Gymnastics is an increasingly popular sport and an important event in the Olympic Games. However, the number of unavoidable injuries in sports is also increasing, and the treatment after the injury is very important. We reduce the harm caused by the injury through the identification and research of pictures. Image preprocessing and other methods can in-depth learn about gymnastics sports injuries. We identify the injured pictures of athletes to know the injury situation. Through the analysis of the force of the athletes during exercise, they can be better integrated into picture recognition for sports injuries. More appropriate prevention and treatment measures are suggested.

## 1. Introduction

The purpose of this article is to recall NCAA monitoring data on women's gymnastics injuries and to identify areas of potential injury prevention measures. Between 1988 and 1989, 1,550 people participated in the women's varsity gymnastics team. In 2003, the number of varsity teams decreased, affecting many participants. The results showed that the average annual injury rate during the sample period decreased, but not in practice. In 16 years, the injury rate in the competition was higher than the actual rate, and the lower half of the body was injured more frequently. The reason for the injury was explained, and suggestions were made for those with bare feet. Athletes use prophylactic tape to reduce the risk of injury, and preventative measures will add more planning to training to enhance the correct mechanism and make the device more absorbent [1]. Sports trainers collected injury information from many participants and looked at a wide variety of injuries. Compared to other sports, injuries to the back and lumbar regions were more frequent, and bare feet injuries were also common because the thigh and the leg were overused; it can be seen from the above that women's gymnastics has great room for improvement in many aspects [2]. This article examines the number and types of injuries in non-professional gymnasts. At the same time, it can also know what are most dangerous, and investigate whether various factors such as the proportion of participants' coaches affect the injury rate. Survey data show that professional gymnasts have higher injury rates and non-athletes have lower rates. To sum up, the level of competition has a great relationship with the injury rate [3]. College-level injury testing data are limited. The researchers still have not caught non-time-loss injuries. The purpose was to describe the epidemiology of injuries in women's gymnastics by pooling injury and exposure data from 11 sports. Athletic data, injury rates, injury rates for body parts, diagnoses, and equipment for collegiate athletes participating in women's gymnastics were derived from 28 seasons of data [4]. Long-term exercise for youth training and competitions can lead to back pain that must not be relaxed. Measures should be taken to treat it when one is young, and treat it in the early stage to achieve a higher therapeutic effect. For pain that lasts for a long time, one should pay attention to the need for careful inspection, and bone scans and other techniques should be performed if necessary. Diagnose the cause [5]. Gymnastics has a lower frequency of injuries in sports. At the same time, based on objective information such as gender and age, the pattern of injury can be predicted. Girls are more likely to be injured. Most of them have wrist pain and low back pain, while some spinal abnormalities were also found. Imaging can also guide the recovery of injured athletes, and parents should know that recreational gymnastics has fewer injuries while more injuries have been found in high-level athletes [6]. According to sports clinic observations, patellar joint pain syndrome is higher, sprains are the biggest problem in emergency surgical treatment, overuse is related to the training intensity, and motor skills can also lead to serious leg injuries [7]. Michelle is the club's project manager. With a large number of gymnasts, Michelle is an integral part of the national team plan, who has been training athletes and has produced many gold medal winners. Michelle has an excellent educational background and wrote the Gymnastics School of Excellence program. Michelle will talk about the prevalence of injuries to better care about the growth of youth sports [8]. Infrared images have many advantages, which can make up for the deficiencies of infrared image face clarity. This technology is an important research direction in the field of face recognition. On the basis of analyzing the characteristics of infrared images, it also analyzes humans. The characteristics of a facial recognition software and a new infrared image recognition method are proposed. Experiments show that this method is feasible [9]. Image recognition methods require a large number of samples to achieve good performance. In some cases, a large number of samples cannot be obtained, which will lead to poor performance. However, commonly used neural network classifiers minimize empirical risk. This paper establishes a method to combine wavelet giant and svm by finding the optimal solution. In the simulation, various tank images are extracted and identified separately. The results show that the algorithms that combine the two are better in small sample cases [10]. The correlation matching algorithm is of great significance, but also faces a key problem. The problem is with sudden changes in the image. This paper examines different application areas and investigates new methods to solve the problem. One is to rotate and rebuild new model diagrams under laboratory conditions. Another approach is to exploit fixed features along with image variance and symbolize them as cells of the correlation matching map [11]. Medical images are the research subject, and the characteristics of images cannot be effectively expressed at present. Therefore, in order to improve the recognition rate, a new algorithm is proposed, which combines two kinds of data. The result is that the fused features can be better expressed for medical images, reducing the workload, and decision-level data fusion can achieve higher recognition rates [12]. In image edge distortion correction, the edge fitting is poor, and hence the correction error is large. In this paper, an image edge true correction algorithm based on the equalization algorithm is proposed, the parameters are adaptively switched by the method, and the image is optimized according to the image denoising result. According to the extraction results, the optimization function is obtained. This paper considers the noise barrel distortion and pincushion distortion images as the research objects [13]. In order to meet the requirements, a new hot-stamping image recognition algorithm is proposed. The minimum point is the best result through subsequent operations such as digital image conversion and spatial extraction of channels. The results show that identifying the hot-stamping area and improving the matching speed have guiding significance for the quality detection of this technology [14]. At present, image recognition has research value in the field of computer recognition. With the development of national strength, the application of these innovative technologies to pig detection is of great help to the breeding of animal husbandry and the convenience of human beings. This paper makes a great introduction to the individual identification technology of pigs and the current research direction of this innovative technology [15].

## 2. Image Recognition Research

### 2.1. Image Recognition of Static Objects

We mainly use the following four methods for image recognition: data feature analysis, image preprocessing, feature extraction, and pattern recognition.

#### 2.1.1. Data Feature Analysis

To achieve static object recognition, the data in a specific object image must first be analyzed to determine what data to retrieve in the object image. According to these characteristics, we analyze the characteristics of the image data, choose the appropriate method during the analysis, and find ways to remove the background and highlight the target. From this, we can see that when performing this step, we need to have a comprehensive understanding of the picture, and different methods need to be used for different picture objects.

#### 2.1.2. Image Preprocessing

Because the image will be damaged and polluted by noise, it will more easily become unsuitable for people's needs or lose the essence of the image during transmission and storage. Therefore, we should preprocess the image to reduce its influence on the image. For simpler image recognition systems, image preprocessing usually includes image enhancement and restoration, but for slightly more complex images, we also perform image segmentation on the image.

This step is mainly to weaken the features that people do not need very much to avoid affecting judgment, and at the same time strengthen the features that people need to pay attention to. Spatial domain methods include processing images directly in the spatial domain, which can be divided into two aspects: point operations and neighborhood operations (local operations). The frequency range method only calculates the image transformation value of the specified image transformation area, calculates the image spectrum of the transformed area, and then inverts the final calculated image in the spatial area. The frequency range method is generally divided into high magnification filter, low magnification filter, bandpass filter, and notch filter.

Image restoration is to change the image into its original essence, using prior knowledge to change the process of degrading the image. Image restoration technology needs to establish a degradation model and reverse the degradation process to obtain the optimal image before degradation.

Image segmentation first divides the image into several meaningful regions, then determines whether there are objects of interest in these regions, and narrates the content on the image. At present, we divide image recognition and segmentation into two methods. The first is a region-based image segmentation algorithm and the second is a boundary-based segmentation algorithm. Image segmentation also has its own disadvantages, and image segmentation algorithms have certain limitations in their application. For example, the key to the threshold method is the selection and determination of the threshold. Different thresholds have completely different effects. Setting the threshold too low will produce noise, and setting the threshold too high will eliminate noise. With no noise signal, it is difficult to define the starting points and similarity criteria in region growing methods, and the quality of the adjustments greatly affects the efficiency of segmentation. Edge detection difficulty is a way to balance detection accuracy and noise immunity. The watershed method is too sensitive to noise and thin lines and is prone to over-segmentation. In general, some image segmentation algorithms must target specific types of images or specific applications. Thus far, no general and effective image segmentation algorithm suitable for all image segmentation has been found, and there is no generally accepted objective standard to evaluate segmentation performance.

1. Information acquisition: It is to convert information such as light or sound into electrical information through sensors. Information can be two-dimensional images such as text, images, etc.; can be one-dimensional waveforms such as sound waves, electrocardiograms, and electroencephalograms; or physical quantities and logical values.
2. Preprocessing: It can include A\D, binarization, image smoothing, transformation, enhancement, restoration, filtering, etc., and mainly refers to image processing.
3. Feature extraction and selection: In pattern recognition, it is necessary to extract and select features. For example, a 64 × 64 image can obtain 4096 data, and the original data in the measurement space can be obtained through transformation. Features that reflect the nature of the classification process are feature extraction and selection.
4. Classifier design: The main function of classifier design is to determine decision rules through training, such that the error rate is the lowest when classifying according to such decision rules.
5. Classification decision: Classify the recognized objects in the feature space.

### 2.2. Research Status Based on Image Recognition Technology

Image recognition technology uses computer technology to realize the recognition function of human vision, i.e., to detect and recognize objects from images and other information. Most of the applications of recognition technology in modern augmented reality systems are computer vision-oriented image recognition, including sign-based augmented reality and unsigned augmented reality. The basic principle of augmented reality based on symbol recognition is to solve the current pose of the camera by performing image recognition on the symbol points of fixed geometric figures using ARToolKit, ARTagl, etc. However, augmented reality systems based on sign recognition are narrower in scope, as signs must be within the camera's range. Meaningless augmented reality based on natural characteristics is the application of image recognition technology in augmented reality systems. The feature is that there is no need to arrange panels in advance, and the application scenarios are wide. In 1999, Neunn et al in the University of Southern California began to combine in-image target recognition and tracking algorithms to propose an augmented reality system for scene annotation based on industrial 3-D images.

## 3. Muscle Model

### 3.1. Muscle Model

One of the most important tasks in sports biodynamic farming is selecting the correct muscle model. The most basic muscle model consists of springs and cushioning materials. The muscle model class proposed by Hill in 1938 reflects a single viscoelastic property of muscle. The ground model consists of linear and viscoelastic elements, and the model has been widely used since then for biomechanical analysis. The hedgehog muscle model consists of three elements: an active contraction element, a parallel elastic element, and a series of elastic elements. Among them, the active contraction element is described by three relational expressions: longitudinal tension ratio, velocity tension ratio, and muscle activation level. Muscle activation levels range from 0 (disabled) to 1 (fully activated). When a muscle becomes fully tonic, both muscle strength and muscle fiber length are within a certain range, indicating a “parabolic” relationship. Maximum contractile force occurs at -N. Here death is the optimal length of muscle fibers.

The active force-length curve of a muscle is described by the following parabolic function:

(1) $$\begin{array}{c} \int_{L}^{\text{act}}=1-{\left[\frac{\left(L^{M}/L_{0}^{M}\right)-1}{0.5}\right]}^{2}. \end{array}$$

In this formula, ∫_*L*_^act^ is the length tension relationship when main power is supplied; *L*^*M*^is the muscle length; and *L*_0_^*M*^ is the relatively optimal muscle fiber length. The passive dynamic length curve of the muscle is described below.

The relationship between passive force and length:

(2) $$\begin{array}{c} \int_{L}^{pa}={\left(\frac{L^{M}}{L_{0}^{M}}\right)}^{3}\exp\left(8\frac{L^{M}}{L_{0}^{M}}-12.9\right). \end{array}$$

The relationship between force and velocity can be represented by a hyperbola when the muscle is coaxially isometrically contracted:

(3) $$\begin{array}{c} \int_{v}=\frac{v_{0}^{M}-v^{M}}{v_{0}^{M}+cv^{M}}. \end{array}$$

*v*_0_^*M*^ and *v*^*M*^ represent the speed of retraction and contraction, and in off-axis isometric contraction; the relationship between force and speed is defined as:

(4) $$\begin{array}{c} \int_{v}=\frac{2v_{0}^{M}-b^{\prime }+v^{M}\left(a^{\prime }/F_{0}^{M}\right)}{v_{0}^{M}-b^{\prime }}. \end{array}$$

We assume that the contraction speed of the muscle will be little affected by the viscous effect of the muscle, while the tension speed curve and the tension length N line of the active force of the muscle both need to be scaled by the activation level a (*t*) before they can be used, and there is feathering angle:

(5) $$\begin{array}{c} a=\text{arctg}\left(\frac{L_{0}^{M}\sin\;\partial_{0}}{L^{MT}-L_{S}^{T}}\right). \end{array}$$

*L*^*MT*^=*L*^*T*^+*L*^*M*^cos  *∂*_*L*_^*MT*^ is the total length of the muscle-tendon component; *L*_*S*_^*T*^ is the relaxed length of the tendon; *∂*^0^ is the feathering angle at optimal fiber length; thus, the total muscle force *F*^muscic^ can be expressed as:

(6) $$\begin{array}{c} F^{\text{muscic}}=F_{0}^{M}\left(f_{L}^{\text{act}}f_{v}a\left(t\right)+f_{L}^{pa}\right)\cos\left(\partial \right). \end{array}$$

The expression of muscle activation level *a*(*t*) is:

(7) $$\begin{array}{c} a\left(t\right)=\frac{\left(u^{2}-ua\right)}{t_{\text{rise}}}+\frac{\left(u-a\right)}{t_{\text{fall}}}. \end{array}$$

*F*_0_^*M*^ represents peak muscle force at optimal muscle length, *u* indicates muscle excitability, and *t*_rise_ and *t*_fall_ represent the ascending and descending processes of muscle activation, respectively.

### 3.2. Huxley Muscle Model

Although the Hill model is widely used, it cannot directly reflect the biochemical process of muscle energy production. The principle of chemistry was cited in 1957, when Huxley developed the Huxley muscle model, also known as the sliding silk model.

Huxley and later Zahalak used the distributed torque approximation method when using the slip-line model. The distributed torque approximation transforms the Huxley model into a series of ordinary differential equations, allowing the method to be solved numerically.

The Huxley–Huxley muscle model accounts for the speed of myofilament connection and separation, the overlapping function of calcium activation (the relationship between the sarcomere strength and the sarcomere length), and the relationship between calcium concentration and the activation level in fibers when describing muscle contraction. In optimization theory, the frequency of muscle stimulation is a variable that affects the magnitude of the total muscle force. The frequency range for muscle stimulation is < 1100>124 Hz. The Huxley muscle model is based on the sliding filament theory and describes the biochemical effects of muscle during the contraction process. The distribution function *n*(*θ*) represents the distributed number of cross-bridge connections, i.e., the logarithm of actin and myosin bonds as a function of cross-bridge length. Since bridging and breaking are assumed to be time-dependent functions *n*(*θ*, *t*), the muscle force velocity curve becomes an implicit function. The connection rate *f* and the disconnection rate *g* of the cross-bridge depend on the normalized cross-bridge length *θ*.

(8) $$\begin{array}{c} f\left(\theta \right)=\left\{\begin{array}{c} 0-\infty <\theta <0\\ 43.3\theta 0<\theta <1\\ 01<\theta <\infty \end{array}\right\},\\ g\left(\theta \right)=\left\{\begin{array}{c} 209-\infty <\theta <0\\ 10\theta 0<\theta <1\\ 10\theta 1<\theta <\infty \end{array}\right\}. \end{array}$$

The muscle force-length relationship is included in the overlap factor *∂*(*l*) middle:

(9) $$\begin{array}{c} \partial \left(l\right)=\left\{\begin{array}{c} 1-6.25{\left(l-1\right)}^{2}l<=1\\ 1-1.25\left(l-1\right)l>1 \end{array}\right\}, \end{array}$$

*l*=*l*_*s*_/*l*_*s*,opt_, where *l*_*s*,opt_ corresponds to the sarcomere length when the sarcomere can provide maximum muscle force.

The distribution function *n*(*θ*, *t*) can be expressed as:

(10) $$\begin{array}{c} \frac{\partial n\left(\xi ,t\right)}{\partial t}-v\left(t\right)\frac{\partial n\left(\xi ,t\right)}{\partial \xi }=r\left(t\right)f\left(\xi \right)\left[\alpha \left(l\right)-n\left(\xi ,t\right)\right]-g\left(\xi \right)n\left(\xi ,t\right). \end{array}$$

### 3.3. Image Recognition Digitization

To process images on a computer, we convert the processed analog images into digital image information. Image digitization refers to the sampling and quantization of images, i.e., converting continuous image signals into discrete digital signals for computer processing.

Sampling is the process of discretizing (coordinates) the real space scene to form a digital representation (i.e., the image is represented by the gray value of some points in space, and these points are called sampling points).

A black and white image can be viewed as a two-dimensional continuous function *f*(*x*, *y*), and its value is expressed as the (*x*, *y*) brightness of the location image. A compute digital image is represented by a matrix or two-dimensional matrix [*f*]_*m*×*n*_. Matrix operation is performed on the digital image to obtain the desired image from a two-dimensional continuous function *f*(*x*, *y*) to digital image matrix [*f*]_*m*×*n*_. It involves taking the function values of different data as samples, and using the discrete values that can be obtained to represent the two steps to complete the picture.

Sometimes, the original information of the image obtained after scanning cannot be preserved, i.e., the spatial density of the sample is not suitable or the brightness of the sample is not enough in scale, and the image must be reconstructed from the restoration.

Sampling is the first step in image digitization. In digital images, samples should be taken from two spatial directions. The matrix is obtained by taking *M* along the *x*-direction of the image and N points along the *y*-direction. After a sample has been extracted from an image, the value of the resulting sample must be determined before entering it into a computer. Specifically, span a range of sample values and then use a single value to represent all the values of that layer. According to the integer storage convention in the computer, the value range of the sample value can be divided into *k* =  2^*i*^ level, generally *i* = 8, 7, 6, and the gray pixel value can be divided into 64, 128, 256 levels, generally called 64, 128, 256 grayscale. The higher the number of layers, the closer the actual image retrieved by the quantized value of the sample is to the original image.

Image reconstruction is the inverse process of image sampling done from image *f*_*s*_(*x*, *y*) to consecutive images *f*(*x*, *y*). The commonly used quantization scheme is uniform, namely the length of the sub-cycle. When the sampling theorem is satisfied, we have:

(11) $$\begin{array}{c} F\left(u,v\right)=\Delta x\Delta yF_{s}\left(u,v\right)H\left(u,v\right), \end{array}$$

which is:

(12) $$\begin{array}{c} f\left(x,y\right)=\Delta x\Delta yh\left(x,y\right)*f_{s}\left(x,y\right),\\ H\left(u,v\right)=\left\{\begin{array}{cc} 1u\in \left[-w_{u},w_{u}\right], & v\in \left[-w_{u},w_{u}\right]\\ 0, & \text{other}. \end{array}\right. \end{array}$$

Thus,

(13) $$\begin{array}{c} h\left(x,y\right)=4w_{u}w_{v}\sin\;c\left(2\pi w_{u}x\right)\sin\;c\left(2\pi w_{v}y\right). \end{array}$$

However,

(14) $$\begin{array}{c} f\left(x,y\right)=k\;\sin\;c\left(2\pi w_{u}x\right)\sin\;c\left(2\pi w_{v}y\right)*f_{s}\left(x,y\right). \end{array}$$

Again,

(15) $$\begin{array}{c} f_{s}\left(x,y\right)=\overset{+\infty }{\underset{m=-\infty }{\sum }}\overset{+\infty }{\underset{n=-\infty }{\sum }}f\left(m\Delta x,n\Delta y\right)\delta \left(x-m\Delta x,y-n\Delta y\right). \end{array}$$

The above derivation shows that the reconstructed image is the result of the weighted summation of many two-dimensional sinc functions located on *x*=*m*Δ*x*, *y*=*n*Δ*y*.

One-dimensional case:

(16) $$\begin{array}{c} f\left(x\right)=k\sum_{m=0}^{M-1}\sin\;c\left[2\pi w_{u}\left(x-m\Delta x\right)\right]f\left(m\Delta x\right). \end{array}$$

The essence of sampling is how many points are used to describe an image, and the quality of the sampling results is measured by the image resolution mentioned above. Quantization refers to the range of values to be used to represent each point after the image is sampled. The result of quantization is the total number of colors that the image can hold, which reflects the quality of the sampling. The amount of image data obtained after digitization is very huge, and coding technology must be used to compress the amount of information. In a certain sense, coding and compression technology are key to realize image transmission and storage. There are many mature coding algorithms applied to image compression. The most commonly used are image prediction coding, transform coding, fractal coding, and wavelet transform image compression coding.

Quantization is the process of converting the corresponding continuous change interval of brightness on the sampling point into a single specific number. After quantization, the image is represented as an integer matrix. Each pixel has two properties: position and grayscale. Positions are represented by rows and columns. Grayscale is an integer representing the lightness and darkness at that pixel location.

### 3.4. Image Preprocessing

In the process of image acquisition, due to the influence of signal transmission, camera, brightness, etc., the acquired image will have great noise suppression. In the case of restoring most of the original image, suppress the image noise as much as possible.

Bilateral filtering is a nonlinear filtering method that expresses compromise processing by combining the spatial proximity of images and the similarity of pixel values.

Usually we use: mean filter, median filter, maximum and minimum filter, bilateral filter, and guided filter.

Since the spatial information and grayscale similarity are considered, the purpose of edge preservation and denoising can be achieved. It's native, non-iterative, and simple. The biggest advantage of bilateral filters is that they preserve edges. Since the algorithm in this paper needs to analyze the motion characteristics of gymnastics contours and preserve boundary information, bilateral filters are selected as the image preprocessing method. Bilateral filter is a nonlinear filtering method, which is a compromise processing combining the spatial proximity of the image and the similarity of the pixel value.

The expression for a noisy image is:

(17) $$\begin{array}{c} g\left(x,y\right)=f\left(x,y\right)+n\left(x,y\right), \end{array}$$

where *f* refers to the image after noise reduction and *n* is the noise. The pixel values of the image restored by the bilateral filter are obtained by the method of local weighted average:

(18) $$\begin{array}{c} \overset{⌢}{f}\left(x,y\right)=\frac{\sum_{\left(i,j\right)\in S_{x,y}}w\left(i,j\right)g\left(i,j\right)}{\sum_{\left(i,j\right)\in S_{x,y}}w\left(i,j\right)}. \end{array}$$

*S*_*x*,*y*_ is a neighborhood representing the size of the center point A, and *g*(*i*, *j*) represents each pixel in the neighborhood.

(19) $$\begin{array}{c} w_{s}\left(i,j\right)=e^{\left|{\left(i-x\right)}^{2}+{\left(j-y\right)}^{2}\right|/2\delta_{s}^{2}},\\ w_{r}\left(i,j\right)=e^{{\left|g\left(i,j\right)-g\left(x,y\right)\right|}^{2}/2\delta_{r}^{2}},\\ w\left(i,j\right)=w_{s}\left(i,j\right)w_{r}\left(i,j\right), \end{array}$$

*w*_*s*_ represents the spatial proximity factor and *w*_*r*_ represents the luminance approximation factor. The bilateral filter is affected by three parameters: the filter half-width N, and parameters *δ*_*r*_ and *δ*_*s*_. The larger the N, the stronger the smoothing effect, *δ*_*s*_ and *δ*_*r*_; then, control the attenuation degree of the spatial proximity factor and the luminance approximation factor, respectively.

*w*_*s*_ represents the spatial proximity factor and *w*_*r*_ represents the luminance approximation factor. They determine the smoothing effect of image preprocessing. Control the attenuation of the spatial proximity factor as well as the luminance proximity factor.

When we identify objects, the most critical factor is the gradient (a lot of feature extraction, SIFT, HOG, etc. are essentially the statistical information of the gradient); the gradient is the edge and is the most essential part; to calculate the gradient, naturally grayscale images are used. The color itself is very easy to be affected by factors such as light, and there are many changes in the color of similar objects. So the color itself is difficult to provide key information. 2010PAMI has some work of colorSIFT, which is also the gradient of different channels.

## 4. Analysis of the Experimental Research

### 4.1. Comparison of the Research Methods

**Figure 1:**
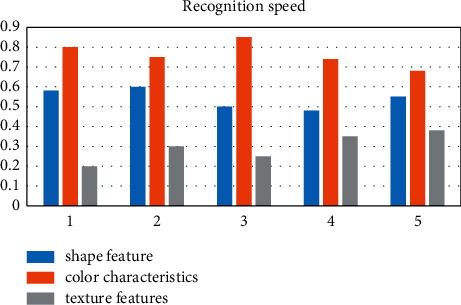
Comparison of the recognition speed.

**Figure 2:**
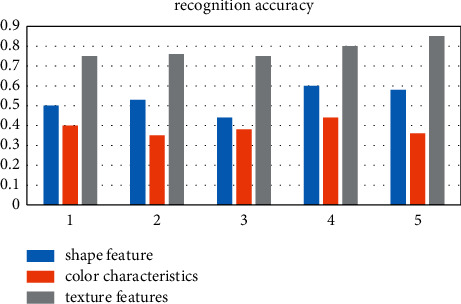
Comparison of the recognition accuracy.

**Figure 3:**
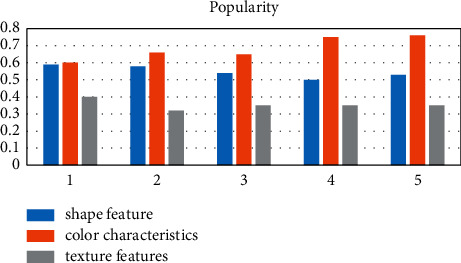
Comparison of the recognition promotion degrees.

There are various methods for image model recognition. According to the features extracted from image pattern recognition, image recognition methods can be divided into the following categories: feature-based shape recognition technology, color feature-based recognition technology, and texture feature-based recognition technology. Among them, the graph-based learning method finds and describes the shape of the object in the image and completes the classification of different images. The common variables that are used to represent shape include parameters, area, circularity, eccentricity, etc. Color detection technology is mainly based on color images, and the color histogram is relatively simple and insensitive to image size, rotation, and type recognition. To detect texture features, various methods that analyze the statistics of very regularly structured lines in the image or the distribution of color intensity in the image are used as shown in Figures 1–3.

Sports injury pictures need more detailed methods to identify; we usually use texture features to identify sports injuries.

After investigating five sets of data, we can clearly see that the three commonly used image recognition methods have their own advantages and disadvantages. In terms of promotion, the public is accustomed to distinguishing from color first; thus, the color features are the most popular, and texture features are the most rigorous and accurate. The rate is the highest, and the shape features are in between.

According to the difference of pattern features and decision patterns, the types of image recognition patterns can be roughly divided into two categories: statistical methods (decision theory) and syntactic (structural) thinking methods. In addition, with the continuous research of pattern recognition technology in recent years, the fuzzy pattern recognition method and the neural network pattern recognition method have also been widely used.

Various retrieval methods based on shape features can effectively use the target of interest in the image for retrieval, but they also have some common problems, including: ① The current retrieval methods based on shape still lack a relatively complete mathematical model; ② If the target is deformed, the retrieval results are often unreliable; ③ Many shape features only describe the local properties of the target, and a comprehensive description of the target often requires high computing time and storage capacity; ④ Many shape features reflect that the target shape information is not completely consistent with human intuition, or the similarity in the feature space is different from the similarity perceived by the human visual system. In addition, the 3D object represented from the 2D image is actually just the projection of the object on a certain plane in space. The shape reflected from the 2D image is often not the real shape of the 3D object, due to the change of viewpoint, and various distortions may occur.

It can be clearly seen that the three commonly used image recognition methods have their own advantages and disadvantages. In terms of promotion, the public is accustomed to distinguishing from color first, so color features are the most popular, texture features are the most rigorous, the recognition accuracy is the highest, and shape features are the highest in between.

### 4.2. Research Objects and Methods

#### 4.2.1. Research Objects

The famous rhythmic gymnasts participating in the “China Art Sports Cup” National Rhythmic Gymnastics Championship are shown in Table 1.

#### 4.2.2. Research Methods

We use sports injury registration in medical research institutes and perform pathological picture recognition on gymnasts. Sports injury identification is related to physical health. We adopt a more rigorous texture identification and mainly investigate the injury situation in the past year.

**Figure 4:**
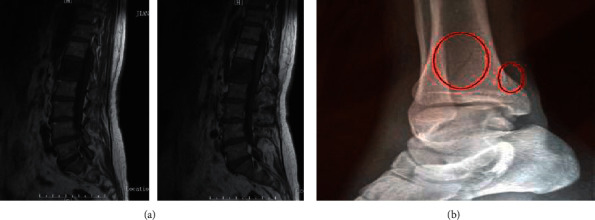
Damage site map. (a) Gymnast spinal injury image and (b) Gymnast foot naked sprain image.

The above Figure 4 is an example of the injuries caused by gymnastics. We can judge the severity of the injury by performing texture recognition on the pictures and come up with a better treatment plan. After more than 100 case studies, the research results were obtained.

### 4.3. Research Results

#### 4.3.1. Overall Incidence of Sports Injuries

During the survey, we identified the pathological pictures of the sports injuries of 126 athletes to determine the injuries. The survey results showed that only a small number of athletes were not injured, and the injury rate was as high as 80%. See Table 2 below.

#### 4.3.2. Injury Nature of Sports Injuries

We investigated the injury nature of sports injuries, and the results showed that a total of 172 cases (person) had mild or severe injuries in sports. Our survey results are shown in the following Table 3. (See Table below).

#### 4.3.3. Injury Degree of Sports Injuries

We investigated the injury degree of sports injuries and found that the degrees of sports injuries were different. Mild injuries accounted for 84.30% of the surveyed proportions, moderate injuries accounted for 14.53%, and severe injuries accounted for 1.16%. See Table 4.

#### 4.3.4. Injured Tissue of Sports Injuries

We surveyed 172 injured athletes and found that there were 0 skin injuries, accounting for 0% of the total injuries; 84 injured athletes had muscle injury, accounting for 48.84% of the respondents, 35 injured athletes has joint injury, accounting for 20.35% of the respondents, 52 people had skeletal injury, accounting for 30.23% of the respondents, and 1 person had nerve injury, accounting for 0.58% of the respondents. See Table 5.

#### 4.3.5. Injury Site of Sports Injury

Rhythmic gymnasts were injured in the following order: spine (68), bare feet (45), lower limbs (30), knee joint (20), shoulder (3), wrist (2), elbow (20), head (1), and chest and abdomen (1). See Table 6.

#### 4.3.6. Injury Causes of Sports Injuries

According to consultation documents, expert opinions, and conversations with athletes, techniques, drawbacks, equipment, designated venues, poor physical conditions, insufficient training activities, chronic excessive exercise, inappropriate training methods, etc. are the main causes of injury. A questionnaire based on the same causes was circulated to the athletes. There are 10 types of damage from fatigue, and one or more causes of damage can be selected for each type of damage. The causes of injury were classified as: prolonged excessive exercise (78 cases), improper training methods (38 cases), technique (23 cases), fatigue (14 cases), inertia (10 cases), insufficient preparation (10 cases), and many other specific reasons. See Table 7.

#### 4.3.7. Treatment and Prognosis of Sports Injuries

In this survey, many athletes use traditional Chinese techniques such as acupuncture, massage, cupping, scraping, etc. for treatment, and some also use stretching, fixed reduction, and strength training physiotherapy for rehabilitation. The specific survey results are shown in Table 8. Athletes feel that the treatment effect is not obvious.

### 4.4. Analysis of the Research Results

#### 4.4.1. Overall Incidence of Sports Injuries

**Figure 5:**
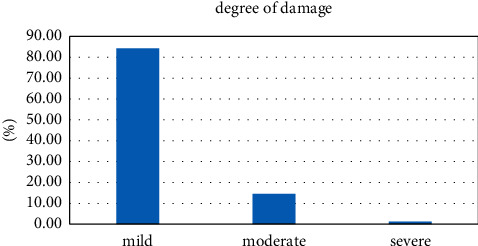
Incidence of rhythmic gymnastics injuries.

As shown in Figure 5, this study investigated 126 rhythmic gymnasts participating in the “China Rhythmic Sports Cup” National Gymnastics Championships. Among them, 102 athletes had injuries of varying degrees. The high incidence of total injuries is a huge risk for the development of rhythmic gymnastics in our country. It can be seen that in the development process of gymnastics, more attention should be paid to sports injuries and the active measures and the effectiveness of preventive measures to prevent or reduce the occurrence of athletes' injuries as much as possible.

#### 4.4.2. Injury Nature of Sports Injuries

**Figure 6:**
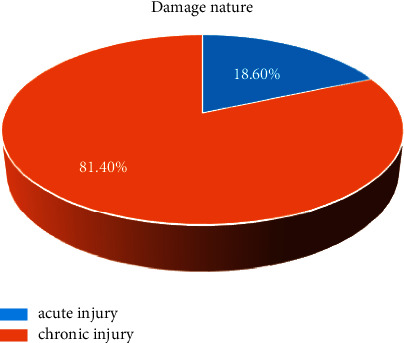
Damage nature.

The ratio of acute to chronic injuries in a survey of 172 injuries is shown. We found that chronic injuries in gymnastics are much higher than in other sports. Chronic injuries are becoming more common as training intensity increases the physical wear and tear on the body. As with most competitive sports, chronic injuries in rhythmic gymnastics are more common than in other sports. The incidence of chronic injuries has gradually increased due to the increased technical difficulty and increased exercise duration, load, and intensity in addition to non-systematic rehabilitation after acute injury, training with injury, and chronic injury. This suggests that solutions for chronic injury and post-injury training and rehabilitation are still insufficient as shown in Figure 6.

#### 4.4.3. Injured Tissue of Sports Injuries

**Figure 7:**
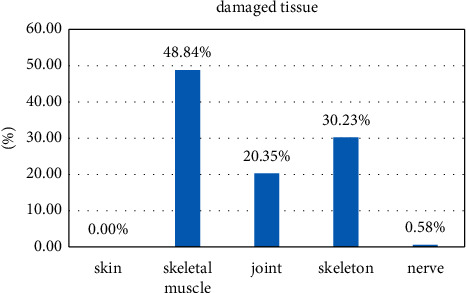
Damaged tissue.

As shown in Figure 7, among the 172 injuries in this investigation, the proportion of injured tissue in each athlete in sports injuries is shown in the figure. Obviously, we can conclude that muscle and spinal cord injuries and keen injuries are due to gymnastics and cause polyrhythmic damage. Skeletal muscle injuries were particularly common, suggesting that problems with post-exercise muscle strength and rhythm recovery should be of great concern to gymnasts.

#### 4.4.4. Injury Site of Sports Injury

**Figure 8:**
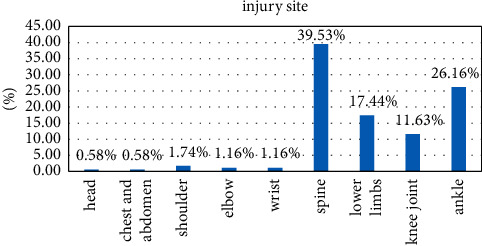
Injury site.

Gymnastics sports injuries mainly include three injured parts: the spine, ankles, and lower limbs, as shown in Figure 8, which corresponds to the characteristics of rhythmic gymnastics. Gymnast rhythm is a fighting art that requires various combinations of movements, such as balance, dance, rotation, and similar movements; the spine is the center of the trunk activity, and the muscles are the driving force for spinal movement. Spinal cord injuries include cervical, thoracic, lumbar, the five segments of the coccyx, the muscle and fascia of the vertebral lobe, and the upper vertebral body. The rhythm of gymnasts requires athletes to have good spinal flexibility, strong muscle strength, and three-dimensional dynamics to perfect difficult and complex movements. Rhythmic gymnastics and gymnastics have excellent movements, fast speed, and many darts: if the strength is insufficient, the movements are fierce, and it is easy to cause local cumulative damage. The spinal muscles are rich and different in size and degree; a lack of strength or imbalance results in spinal deformities, such as cervical vertebral arch and thoracic scoliosis. Investigations have shown that the curvature of the spine in athletes is very pronounced and to varying degrees; the deformation of the spine in turn affects the center of motion of the entire chain, and then a vicious circle begins, affecting most disciplines. In the past, spinal cord injury was much larger than knee joint and other joint injuries, accounting for 39.53% of the total injury, which promoted the solution of the prevention and treatment of spinal cord injury. The spine is the central axis and pillar of the human body and has the function of supporting the load; the spine is an arched structure with good elasticity, which plays the role of transmitting pressure and damping vibration. The lever is attached to this point. The nature of this phenomenon promotes the uneven development of muscle strength on both sides of the spine, resulting in twisting of the spine, which in turn leads to a series of injuries to the bones, muscles, and fascia. This is a vicious circle that seriously affects the sports discipline and competition. The muscles on both sides of the spine are very rich and are critical to the balanced development of muscle strength in addressing spinal cord injuries. Core strength training reduces spinal cord strain, thereby reducing the damage to various spinal structures. While strengthening both sides, the weak side should be lifted, but the extension of strength should not been neglected. Deep muscles should be strengthened via exercise; the stability of the spine should be strengthened and the physiological structure of the spine should be normalized. In terms of recovery training, therapeutic treatment, spinal manipulation, spinal gun application, and extension, treatment, and rehabilitation methods are coordinated and intersected to achieve the purpose of spinal cord injury rehabilitation. The reason for ankle injuries is that rhythmic gymnastics is a sport based on dance equipment (rings, balls, sticks, belts, and ropes); standing movements are usually done by steps and by weight. In addition, when jumping, balancing, and turning around, the center of gravity of the human body changes, and the gravity falls on the outermost side of the foot, causing uneven force on the swinging part of the foot. The muscles and ligaments on the restless side are prone to cause ankle flexion and foot inversion, joint dislocation, and damage the lateral synovial joint and the anterior fibular ligament. If there is no systematic rehabilitation training, it is easy to hurt the ankle. The correct rhythm of gymnastics is to walk in “beauty” with both feet. Therefore, the form of external opening is necessary, which has a great relationship with the gymnastics rhythm, and the external rotation of the lower limbs must reach the maximum external hip. Herman's axis is always in a straight line. However, “enlightened measures must be taken to grip the joint so that the inside of the foot touches the ground and ‘falls the foot'.” The high incidence of disc injuries in the foot cannot be underestimated and often affects whether a player is able to train and play. Important: A support band or ring can be applied to the swinging joint prior to exercise to reduce or prevent injury around the joint to be repaired. In addition to structural normalization, tissue rehabilitation must incorporate the complex functional capabilities of this part. The lower extremity is primarily fatigued (periostitis), often doing standing kicks and jumping exercises; fatigue is easily caused by the anterior osseous muscles, and these muscles are not easy to stretch and relax at will. Periostitis is a result of fatigue. The main reason is that there are too many technical jumps but insufficient knee muscle strength, difficult training, unreasonable technical movements, etc. In summary, we can clearly see that young gymnasts have limited physical ability and their physical ability is affected by fiber load. The coach must follow and guide patiently, train step by step, and try to avoid sports injuries.

#### 4.4.5. Sports Injury Factors

**Figure 9:**
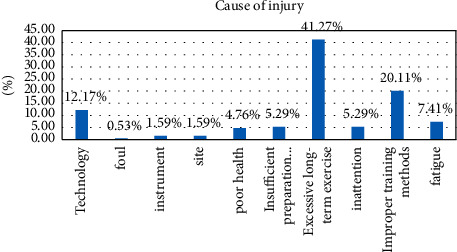
Causes of damage.

See Figure 9. Among the injury factors, too long training time, improper training methods, and technical reasons are the main injury factors of sports injuries, and preparations for the athlete's physiological and physical functions, medical monitoring, and follow-up care should be made at all times. The training volume, excessive training, and fatigue can make the athlete's body more prone to injury. Coaches should have a more comprehensive understanding of each athlete and arrange training reasonably according to each athlete's situation and should also work harder on the prevention of fatigue.

Fatigue and physical conditions can lead to injuries, suggesting the need to establish precautionary principles. In addition, the lack of preparation and laziness are also completely preventable causes of injury. Coaches should always remind athletes to prepare carefully and fully participate in warm-up exercises, so as to better protect their bodies and devote themselves to training.

#### 4.4.6. Treatment and Prognosis of Sports Injuries

Injury cases in this investigation were treated by the following methods: massage, acupuncture, bandages, sealants, strength training, foot baths, stretching, cupping, ice compresses, scraping, repositioning, limbs, acupuncture, braking, nutritional medicine, and physiotherapy (mainly intermediate frequency current and ultrashort wave). From the above statistics, it can be seen that most injuries are treated by traditional methods. Acupuncture and massage methods such as active position, tape, ice pack, physiotherapy, rehabilitation training, and other methods still account for a small proportion of the damage. A majority of the athletes who participated in the survey indicated that the treatment methods are effective in relieving pain from acute injuries, but injuries are likely to recur frequently. Complete rehabilitation of the injury site is important in addition to its anatomy to restoring normal tissue and tissue representation. The recovery of this part of complex functional ability requires muscle groups to restore the ability to work in coordination and precision work, and current injury treatments do not include rehabilitation training. It ignores the thorough treatment of the injured party. When this happens, repeat exercise can lead to the accumulation of injuries and gradually step into chronic injuries. Controlling the wearing of joint support belts through the necessary fastening techniques of support belts and the wearing of protective equipment is an important and effective method to protect knee joints and prevent joint injuries. Application of knee pads can protect the knee joint from the impact force after landing. The application of intramuscular action has a good effect on preventing muscle and joint damage. Therefore, it is necessary for us to increase the use of bandages in rhythmic gymnastics to reduce the occurrence and aggravation of injuries. Strength training includes specific muscle strength training, small muscle group strength training, and core strength training, Core strength training can significantly alter the core stability of rhythmic gymnasts. However, access to scientific training requires good medical supervision. Support belts are used for damaged areas.

Taking preventive measures can help reduce the incidence of injuries, such as exercising before exercising, wearing protective equipment, and performing warm-up activities, which can help reduce the incidence of injuries. After training, athletes can completely relax in various ways, especially active stretching and athlete relaxation, which will not only allow athletes to relax completely, but also improve the flexibility of the athletes' whole bodies and make difficult movement skills go more smoothly; increase the standardization and differentiated treatment of technical gestures during training; effective treatment methods should be adopted in a timely manner after an athlete is injured. Another aspect of scientific training is to monitor the physiological and biochemical functions, and use scientific means to ensure the training level of athletes.

**Figure 10:**
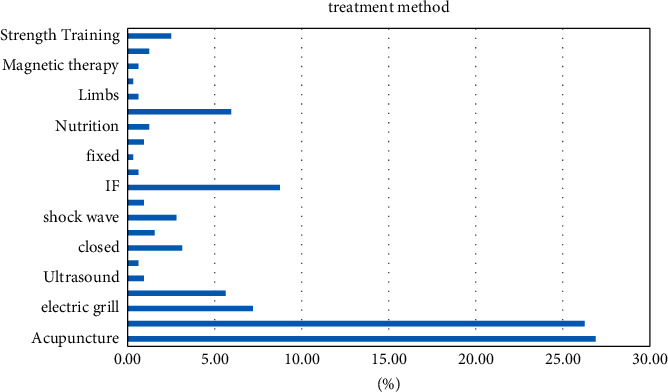
Treatment method.

From this article, we can know that gymnastics injuries are mostly chronic injuries, and the treatment of chronic injuries is mostly massage. In sports injuries, we can see from Figure 10 that massage, acupuncture, and other treatments to restore body function are more effective.

## 5. Conclusion

Through the comparison of various studies and surveys of gymnastics, it can be concluded that the study of injuries in gymnastics is necessary and critical because injuries are inevitable when the general public and professional sportspeople engage in sports. By studying the force analysis of muscle mechanics, we can find the cause of injury during exercise, reduce the probability of injury, and also draw a more effective method of treatment. Our research can better allow the public to participate in gymnastics and reduce the risk of injury. Image recognition research also provides better research conditions for gymnastics sports injury research.

## Figures and Tables

**Table 1:** Basic information of athletes.

*n* = 126	Age (y)	Height (cm)	Weight (kg)	Years of exercise (y)
Woman	15.34 + −2.44	160.39 + −7.18	42.54 + −6.98	6.16 + −2.65

**Table 2:** Incidence of sports injuries.

*n* = 126	Number of injuries	No injuries	Total
Woman (*n* = 126)	102	24	125
Percentage%	80.95	19.05	100

**Table 3:** The nature of sports injuries among athletes in rhythmic gymnastics.

Damage nature	Acute injury	Chronic injury	Total
Visits	32	140	172
Percentage%	18.60	81.40	100

**Table 4:** Degree of sports injuries among athletes in rhythmic gymnastics.

Degree of damage	Mild	Moderate	Severe	Total
Visits	145	25	2	172
Percentage%	84.30	14.53	1.16	100

**Table 5:** Organization of sports injuries among athletes in rhythmic gymnastics.

Damaged tissue	Skin	Skeletal muscle	Joint	Skeleton	Nerve	Total
Visits	0	84	35	52	1	172
Percentage%	0	48.84	20.35	30.23	0.58	100

**Table 6:** Sports injuries of athletes in rhythmic gymnastics.

Injury site	Head	Chest and abdomen	Shoulder	Elbow	Wrist	Spine	Lower limbs	Knee joint	Bare feet	Total
Visits	1	1	3	2	2	68	30	20	45	172
Percentage (%)	0.58	0.58	1.74	1.16	1.16	39.53	17.44	11.63	26.16	100

**Table 7:** Causes of sports injuries among athletes in rhythmic gymnastics.

Cause of injury	Technology	Foul	Instrument	Site	Poor health	Insufficient preparation activities	Excessive long-term exercise	Inattention	Improper training methods	Fatigue	Total
Visits	23	1	3	3	9	10	78	10	38	14	317
Percentage%	12.17	0.53	1.59	1.59	4.76	5.29	41.27	5.29	20.11	7.41	100

**Table 8:** Treatment methods for sports injuries.

Treatment method	Visits	Percentage (%)
Acupuncture	86	26.88
Massage	84	26.25
Electric grill	23	7.19
Dressing	18	5.63
Ultrasound	3	0.94
Reset	2	0.63
Closed	10	3.13
Foot bath	5	1.56
Shock wave	9	2.81
Cupping	3	0.94
IF	28	8.75
Scraping	2	0.63
Fixed	1	0.31
Ice	3	0.94
Nutrition	4	1.25
Ultrashort	19	5.94
Limbs	2	0.63
Small needle knife	1	0.31
Magnetic therapy	2	0.63
Pulling	4	1.25
Strength training	8	2.5

## Data Availability

The experimental data used to support the findings of this study are available from the corresponding author upon request.

## References

[1] Dick R., Romani W. A., Agel J. (2007). Descriptive epidemiology of collegiate women’s gymnastics injuries: national collegiate athletic association injury surveillance system, 1988–1989 through 2003–2004. *Journal of Athletic Training*.

[2] Garrick J. G., Requa R. K. (1980). Epidemiology of women’s gymnastics injuries. *The American Journal of Sports Medicine*.

[3] Lowry C. B., Leveau B. F. (1982). A retrospective study of gymnastics injuries to competitors and noncompetitors in private clubs. *The American Journal of Sports Medicine*.

[4] Kerr Z. Y., Hayden R., Barr M. (2015). Epidemiology of national collegiate athletic association women’s gymnastics injuries, 2009-2010 through 2013-2014. *Journal of Athletic Training*.

[5] Micheli L. J. (1985). Back injuries in gymnastics. *Clinics in Sports Medicine*.

[6] Keller M. S. (2009). Gymnastics injuries and imaging in children. *Pediatric Radiology*.

[7] Andrish J. T. (1985). Knee injuries in gymnastics. *Clinics in Sports Medicine*.

[8] Highden M. D. Gymnastics - sport development and influence on growth related injuries.

[9] Li J., Yu W. X., Kuang G. Y. (2006). Research on face recognition approaches of infrared image. *Journal of National University of Defense Technology*.

[10] Shi J. F., Bei S. Research on image recognition based on invariant moment and SVM.

[11] Tang M. (1996). Research of target image recognition based on correlation match. *Proceedings of SPIE - The International Society for Optical Engineering*.

[12] Song Y. Q., Chen J. M., Guo Y. Z. (2008). Research on multi-feature medical image recognition based on data fusionResearch on Medical Image Recognition Based on Multi-feature Fusion. *Application Research of Computers*.

[13] Fang Q., Zeng W., Liu R. (2021). Research on intelligent image recognition technology based on equalization algorithm. *Journal of Physics: Conference Series*.

[14] Zhang D. J., Tang W. Y. (2014). Research of hot stamping image recognition algorithm based on projection feature. *Applied Mechanics and Materials*.

[15] Gu X., Song H., Chen J. (2020). A review of research on pig behavior recognition based on image processing. *International Core Journal of Engineering*.

